# Carbon Catabolite Repression Regulates the Production of the Unique Volatile Sodorifen of *Serratia plymuthica* 4Rx13

**DOI:** 10.3389/fmicb.2017.02522

**Published:** 2017-12-19

**Authors:** Nancy Magnus, Teresa Weise, Birgit Piechulla

**Affiliations:** ^1^Institute for Biological Sciences, University of Rostock, Rostock, Germany; ^2^EuroImmun, Medizinische Labordiagnostik AG, Lübeck, Germany

**Keywords:** *Serratia plymuthica*, volatile organic compounds (VOCs), sodorifen, terpene, carbon catabolite repression, adenylate cyclase, cAMP receptor protein, carbon catabolite responsive element

## Abstract

Microorganisms are capable of synthesizing a plethora of secondary metabolites including the long-overlooked volatile organic compounds. Little knowledge has been accumulated regarding the regulation of the biosynthesis of such mVOCs. The emission of the unique compound sodorifen of *Serratia plymuthica* isolates was significantly reduced in minimal medium with glucose, while succinate elevated sodorifen release. The hypothesis of carbon catabolite repression (CCR) acting as a major control entity on the synthesis of mVOCs was proven by genetic evidence. Central components of the typical CCR of Gram-negative bacteria such as the adenylate cyclase (CYA), the cAMP binding receptor protein (CRP), and the catabolite responsive element (CRE) were removed by insertional mutagenesis. CYA, CRP, CRE1 mutants revealed a lower sodorifen release. Moreover, the emission potential of other *S. plymuthica* isolates was also evaluated.

## Introduction

Microorganisms have an outstanding potential to survive in very different habitats, environments, and under diverse nutritional conditions. As a consequence, they often produce a plethora of secondary metabolites, including volatile organic compounds (VOCs), which are characterized as small molecular compounds with high vapor pressures, low boiling points and molecular masses below 300 Da (summarized in Schulz and Dickschat, 2007; Effmert et al., 2012; Lemfack et al., 2017). Studies on fungal and bacterial VOCs lag behind the knowledge on plant and animal derived VOCs, e.g., only limited information of biological/ecological roles and modes of actions of these compounds are available (Piechulla et al., 2017) and it often remains unknown under which conditions the compounds are produced and released, and which underlying regulatory circuits control these processes. In fact, the microbial bouquets vary regarding quantity and quality of the small volatile metabolites, which derive from various biosynthetic pathways (e.g., fatty acid derivatives, aromatic compounds, nitrogen-containing compounds, sulfur-containing compounds, terpenoids, and halogenated-, selenium-, tellurium-, and metalloid-containing compounds, Schulz and Dickschat, 2007). While the general principles of the biosynthetic pathways are known for many compound classes, often details of specific reactions and mechanisms as well as involved enzymes remain so far elusive, e.g., 10-methylundecan-2-one of *Xanthomonas campestris* (Weise et al., 2012), 1,2,4,5,6,7,8,-heptamethyl-3-methylene-bicyclo(3.2.1)oct-6-ene (sodorifen) of *Serratia plymuthica* 4Rx13 (Domik et al., 2016a), distinct pyrazines of *Serratia rubidaea* (Kai et al., unpublished results).

The rhizobacterium *S. plymuthica* 4Rx13 emits the unusual volatile compound sodorifen whose underlying biosynthesis remains elusive due to its unusual structure. According to the latest results, sodorifen is a sesquiterpene (Domik et al., 2016a) but nothing is known about the biological function of this volatile. Here we present results concerning the regulation of sodorifen emission, demonstrating for the first time that microbial volatiles are under the control of the carbon catabolite repression (CCR).

Sodorifen is a volatile secondary metabolite with an extraordinary and unique structure (C_16_H_26_), which was elucidated previously (von Reuß et al., 2010). It is composed of a C5- and C6-ring, where every carbon atom of the skeleton is substituted with either a methyl or a methylene group. It was shown that this novel sesquiterpene is only emitted by a few Gram-negative *S. plymuthica* isolates (Weise et al., 2014). However, the catalytic reactions behind this complicated structure are so far unknown, although terpenoids, including sesquiterpenes, are well-known plant secondary metabolites (Pichersky and Raguso, 2016). Microorganisms, most importantly *Actinomycetales*, are also a rich source of terpenes (Yamada et al., 2015; Dickschat, 2016) and the majority of sesquiterpene synthases have been isolated from *Streptomyces* species. Recently, we discovered a unique cluster of four genes involved in the biosynthesis of the sesquiterpene sodorifen, being the first described sesquiterpene synthase gene isolated from *Enterobacteriaceae* (Domik et al., 2016a,b). We noticed that sodorifen comprises ca. 50% of the total VOC spectrum of *S. plymuthica* 4Rx13 (Kai et al., 2010), and the emission was significantly increased when *S. plymuthica* was cultivated in minimal medium supplemented with succinate instead of complex medium (nutrient broth) (Weise and Piechulla, unpublished). So far, nothing is known about the biological function and biosynthetic control of sodorifen. Since such adaptations of the metabolism due to changed carbon supply are often a consequence of altered transcriptional control, we hypothesized that CCR might be involved to control and regulate the emission of the secondary metabolite sodorifen in *S. plymuthica* 4Rx13.

Carbon catabolite repression is a prominent regulatory network where a carbon compound (often glucose) inhibits the synthesis of enzymes involved in catabolism of other carbon sources (e.g., lactose) (summarized in Stülke and Hillen, 1999; Deutscher, 2008; Görke and Stülke, 2008). The underlying regulatory mechanism includes the activation of the adenylate cyclase resulting in increased cAMP levels. Both, cAMP and its receptor protein (CRP or catabolite activator protein CAP) bind to catabolite responsive elements (CREs sequence) upstream of the promoter, supporting RNA polymerase binding and subsequently facilitating transcription. This transcriptional control process addresses particularly genes that encode enzymes involved in the catabolism of carbon compounds of the primary metabolism of bacteria but some examples are also known where CCR controls reactions of the secondary metabolism (Ruiz et al., 2010; van Wezel and McDowall, 2011).

In our study we constructed insertional deletion mutants of central CCR genes, i.e., of the adenylate cyclase (*cya*), the cAMP receptor protein (*crp*) and potential binding sites of the cAMP/CRP complex upstream of the sodorifen cluster (CRE) and thereby demonstrated for the first time that microbial volatiles are under control of CCR.

## Materials and Methods

### Bacterial Strains, Media, and Growth Conditions

All strains used in this study are described in Domik et al. (2016a). Additionally, the isolate from the anthosphere of Styrian oil pumpkin *S. plymuthica* S13 was used (Fürnkranz et al., 2012). Originating from *S. plymuthica* 4Rx13 wild type, several insertion mutants were constructed using homologous recombination (insertional mutagenesis) described in Domik et al. (2016a). The following mutants were constructed: adenylate cyclase *cya*::Km (SOD_c01380; *cya*::FRT-PGK-gb2-neo-FRT, Km^R^), cAMP receptor protein *crp*::Km (SOD_c43810; *crp*::FRT-PGK-gb2-neo-FRT, Km^R^), catabolite responsive element CRE1::Km (CRE1::FRT-PGK-gb2-neo-FRT, Km^R^), CRE2::Km (CRE2::FRT-PGK-gb2-neo-FRT, Km^R^). *S. plymuthica* wild type strains and mutants were cultivated either in complex or minimal medium. NB (Nutrient broth, pH 7.2, SIFIN, Berlin, Germany) was used as complex liquid medium. Through addition of 15 g/l agar-agar solid NB medium was obtained. If necessary, an appropriate antibiotic (e.g., kanamycin, 50 μg/ml; tetracycline 10 μg/ml) was added to the medium. As minimal medium (MM), modified Davis and Mingioli medium (DMM) supplemented with different carbon sources was utilized [7 g/l K_2_HPO_4_, 3 g/l KH_2_PO_4_, 0.5 g/l sodium citrate, 0.1 g/l MgSO_4_ × 7 H_2_O, 1 g/l (NH_4_)_2_SO_4_, pH 6.2; Davis and Mingioli, 1950]. 55 mM sterile filtered succinate or glucose were added as carbon source (Millipore filters, pore size 0.2 μm, Sarstedt, Nümbrecht, Germany). Main cultures were prepared by inoculating 100 ml of liquid medium (final OD_600_ of 0.005) with bacteria of an overnight grown preculture. Cultures were incubated for up to 72 h at 170 rpm and 30 or 37°C.

### Isolation of Genomic and Plasmid DNA

Isolation of DNA was performed from overnight grown precultures or main cultures. Genomic DNA was purified using the NucleoSpin^®^ Tissue Kit (Macherey-Nagel, Düren, Germany) according to the manufacturer’s instructions for bacterial cells. Plasmid-DNA was isolated with the NucleoSpin^®^ Plasmid Easy Pure Kit (Macherey-Nagel, Düren, Germany) following the instructions. DNA concentration and integrity were tested photometrically and by gel electrophoresis. Horizontal gel electrophoresis was performed in 1% (w/v) agarose gels (Carl-Roth GmbH+Co., KG, Karlsruhe, Germany) with 1x TAE buffer (40 mM Tris base, 20 mM acetic acid, 1 mM EDTA) containing 0.7 μg/ml ethidium bromide.

### Isolation of Total RNA

The isolation of bacterial RNA was performed according to a modified protocol for hot-phenol extraction after Oelmüller et al. (1990). For this purpose, cell pellets were harvested from main cultures after 24–48 h incubation by centrifugation (10 min, 10,000 × *g*, 4°C). The pellets were resuspended in 600 μl cold AE buffer (2.72 g/l sodium acetate × 3H_2_O, 0.372 g/l Na_2_EDTA × 2H_2_O, pH 5.5) and subsequently transferred to prewarmed SDS-phenol solution (15 μl 25% w/v SDS in 1.2 ml phenol, 65°C). After incubation for 10 min at 65°C the samples were centrifuged for 45 min at 10,000 × *g* and 4°C. The upper, aqueous phase was transferred into a new tube, followed by addition of 100 μl 2 M sodium acetate and 600 μl phenol with subsequent centrifugation (30 min, 10,000 × *g*, 4°C). Again, the aqueous phase was transferred into a new tube followed by another phenol extraction. The resulting upper phase was diluted in 0.75 volume 8 M LiCl and incubated for 30 min at -20°C, followed by centrifugation (10 min at 10,000 × *g* and 4°C). The pellet was resuspended in 300 μl DEPC-treated, sterile water (DEPC-H_2_O) with subsequent addition of 30 μl 3 M sodium acetate (pH 5.2) and 750 μl cold ethanol. Incubation for 30 min at -70°C was followed by centrifugation (10 min at 13,000 × *g* and 4°C). The RNA pellet was washed in 70% v/v ethanol, then dried and suspended in 40 μl DEPC-H_2_O. RNA concentration was determined photometrically and integrity of RNA isolates was checked using denaturing gel electrophoresis in 1% w/v agarose gels prepared with 1x RB buffer (20 mM MOPS, 5 mM sodium acetate, 0.5 mM EDTA) and 3.4% v/v formaldehyde.

### Polymerase Chain Reaction

Polymerase chain reaction (PCR) was performed according to the previously published protocol from Domik et al. (2016a) using either Taq or Phusion^®^ polymerase (Thermo Fisher Scientific, Waltham, MA, United States) and specific primers (Sigma–Aldrich, Munich, Germany; Supplementary Table S1).

### Reverse Transcription PCR

Reverse transcription PCR was used to determine the expression of genes. Isolated RNA was first treated with DNaseI (Invitrogen, Carlsbad, CA, United States) according to the manufacturer’s instructions. Afterward, RNA samples were converted into their corresponding cDNA following the protocol given in Supplementary Table S2. Additionally, a negative control was used by replacing the reverse transcriptase with DEPC-H_2_O to check for potential residual DNA. Finally, the resulting cDNA was amplified using a Taq-based PCR (Domik et al., 2016a) with 2 μl of cDNA as template.

### Northern Blot

For quantitative evaluation of gene expression levels, capillary Northern blot technique was applied according to the protocol for transfer onto nylon membranes at neutral pH, published in Sambrook and Russell (2001). Subsequent labeling of specific RNA molecules was performed with digoxigenin-11-UTP (DIG-dUTP, Roche Diagnostics Deutschland GmbH, Mannheim, Germany)-labeled probes, generated by PCR (Supplementary Table S3). Labeled RNA was detected using anti-DIG antibodies and the substrate CDP-Star (both obtained from Roche Diagnostics Deutschland GmbH, Mannheim, Germany). Visualization and quantification was achieved by chemiluminescence using the imaging system Stella 3200 and AIDA imaging software (raytest, Straubenhardt, Germany).

### Rapid Amplification of 5′ cDNA Ends (5′ RACE)

The determination of the transcriptional start site of the sodorifen gene cluster was conducted using the 5′-RACE technique following the protocol developed by Scotto-Lavino et al. (2006). First, RT-PCR was performed to produce cDNA with subsequent digestion of the RNA template by RNaseH followed by poly-(A)-tailing of the cDNA. Thus, amplification of 5′ cDNA ends was possible using specific primers (GSP1, GSP2, Q_T_, Q_O_ and Q_I_, see Supplementary Table S1) in two successive rounds. Afterward, the result was checked via gel electrophoresis and sequencing.

### Mutagenesis

Site-specific inactivation of genes in *S.p.* 4Rx13 was achieved using the Quick & Easy *Escherichia coli* Gene Deletion Kit (Gene Bridges, Heidelberg, Germany) according to the protocol previously described in Domik et al. (2016a). Thereby, a functional cassette, containing a kanamycin resistance gene (FRT-PGK-gb2-neo-FRT; supplied by helper plasmid pFRT) is inserted into the open reading frame (ORF) of a gene by homologous recombination, causing its disruption. The process of recombination is supported by a helper plasmid (pRed/ET) provided by the kit.

### DNA Sequencing

Sequencing of 5′-RACE PCR products was conducted by GATC Biotech AG (Konstanz, Germany) using Sanger sequencing technique. Previously, DNA constructs to be sequenced were cloned into the pJET1.2 vector (CloneJET PCR Cloning Kit, Thermo Fisher Scientific, Waltham, MA, United States). Primers were provided by GATC (see Supplementary Table S1).

### Sequence Alignments

Alignments of DNA and protein sequences were performed using the Clustal Omega online tool (Sievers et al., 2011).

### Identification of Regulatory Sequences

Potential CRE binding sites were identified with the help of the Regulatory Sequence Analysis Tool for prokaryotes (RSAT; Medina-Rivera et al., 2015) using an already published CRE consensus sequence (Kant et al., 2009) as a motif to search in the 5′ UTR of the *S.p.* 4Rx13 sodorifen cluster.

### Analysis of Volatile Organic Compounds (VOCs)

Bacteria were cultivated overnight in 6 ml liquid medium (170 rpm, 30°C) and subsequently transferred into the VOC collection system (Kai et al., 2010) containing 100 ml medium. Volatiles were collected and trapped on the adsorbent material Porapak (Sigma–Aldrich, Munich, Germany) and eluted in 24 h time intervals using dichloromethane (Carl Roth GmbH+Co., KG, Karlsruhe, Germany) and an internal standard (nonylacetate, final concentration 5 ng/μl). Finally, eluates were analyzed by gas chromatography/mass spectrometry according to the procedure published in Domik et al. (2016a).

## Results

The rhizobacterium *S. plymuthica* 4Rx13 is one of few bacterial strains capable of emitting the new and unusual volatile compound sodorifen. Due to its unique structure, the underlying biosynthesis remains elusive. According to the latest results, sodorifen is a sesquiterpene (Domik et al., 2016a) but still nothing is known about the biological function of this volatile. Here we present results concerning the regulation of sodorifen emission, demonstrating for the first time that microbial volatiles are under the control of the CCR.

### Sodorifen Emission and Expression of the Sodorifen Cluster Genes in *Serratia plymuthica*

**FIGURE 1:**
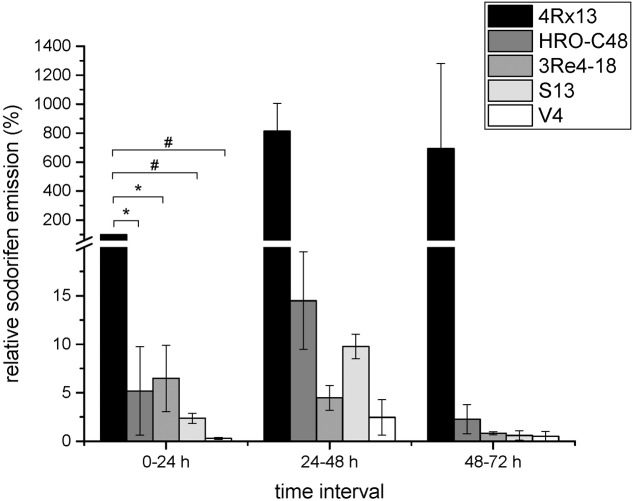
Relative sodorifen emission of the producer strains. The sodorifen emission of the producer strains *S.p.* 4Rx13, *S.p.* HRO-C48, *S.p.* 3Re4-18, *S.p.* S13, and *S.p.* V4 was determined during growth in complex medium using the closed VOC collection system (modified after Kai et al., 2010). Relative sodorifen emission was calculated in relation to the living cell number, with *S.p*. 4Rx13 representing 100% after 24 h cultivation. Error bars indicate standard deviation (*n* = 3). ^∗^*p* < 0.05; ^#^*p* < 0.01.

Five bacterial strains capable of emitting sodorifen were previously identified, including *S. plymuthica* 4Rx13, *S.p.* HRO-C48, *S.p.* 3Re4-18, *S.p.* S13, and *S.p.* V4 (Weise et al., 2014; Domik et al., 2016a). Comparison of the sodorifen amounts emitted by these strains was conducted using the closed VOC collection system followed by GCMS analysis. The bacteria were cultivated in complex medium inside the collection system for 72 h with concurrent elution of the volatiles every 24 h. **Figure 1** presents the relative sodorifen emissions determined in the different producer strains. The results revealed that in *S.p.* 4Rx13, as well as in the other producer strains, the sodorifen emission increased during growth in complex medium until a maximum after 48 h of cultivation was reached, thereafter the emission decreased. Furthermore, it became apparent that at all time intervals *S. plymuthica* 4Rx13 reached the highest amounts of sodorifen emission. Interestingly, all other producers achieved only 0.3–6.5% of the *S.p.* 4Rx13 relative sodorifen emission during the first growth interval (0–24 h). In addition, the relative sodorifen emissions in *S.p.* HRO-C48 and *S.p.* 3Re4-18 were almost equal with values around 5% at 0–24 h and afterward decreased steadily to a minimum of about 0.1–0.3% relative to *S.p.* 4Rx13. *S.p.* V4 produced the least amounts of sodorifen (0.06–0.2%) in relation to *S.p.* 4Rx13. The fact that the sodorifen emission in *S.p.* 4Rx13 exceeded that of the other producers about 15 to 1600-fold indicated a diverging regulation of the sodorifen emission in the different strains tested.

In previous studies a cluster of four consecutive genes was identified in *S.p.* 4Rx13, which is evidently involved in the sodorifen biosynthesis. Furthermore, comparative transcriptome analysis of the sodorifen producer *S.p.* 4Rx13 and the non-producer strain *S.p.* AS9 revealed that this gene cluster was only expressed in the sodorifen-producing isolate (Domik et al., 2016a,b). Due to their similar orientation in the genome of *S.p.* 4Rx13 it was assumed that all four genes are co-transcribed. RT-PCR with various specific primer combinations was performed to amplify the total range of all four sodorifen biosynthesis genes and proved the existence of one large mRNA transcript (Domik et al., 2016b). Since all four sodorifen cluster genes are evidently co-transcribed the results obtained for the terpene cyclase can also be extrapolated for the other three genes of the cluster. The variations in the amount of sodorifen emission by the different producer strains correspond to expression levels of the terpene cyclase detected by RT-PCR (Supplementary Figure S1A) and Northern blot (Supplementary Figure S1B). RT-PCR with primers specific for the terpene cyclase revealed that the sodorifen cluster is expressed in all sodorifen producing strains. Quantification of the expression levels in the Northern blot showed that the strongest signal for the terpene cyclase expression was detected in *S.p.* 4Rx13 (100%). Surprisingly, strong expression of the sodorifen cluster was also detected in *S.p.* 3Re4-18 (129%) despite its lower sodorifen emission. As expected, the other producers (*S.p.* HRO-C48, *S.p.* S13, and *S.p.* V4) showed a weak signal for this gene (ca. 2–18%) in comparison to *S.p.* 4Rx13. Strongest expression for the terpene cylase genes of all producers were obtained in the first growth interval (0–24 h) which included the exponential as well as beginning of the stationary phase, while thereafter all levels decreased until they were no longer detectable (Supplementary Figure S1B). For *S.p.* S13 and *S.p.* V4 no expression could be observed already after 48 h of cultivation, whereas for *S.p.* 4Rx13, *S.p.* HRO-C48, and *S.p.* 3Re4-18 this was only the case after 72 h growth in minimal medium + succinate.

### Analysis of the Sodorifen Cluster 5′-UTR in *Serratia plymuthica*

**FIGURE 2:**
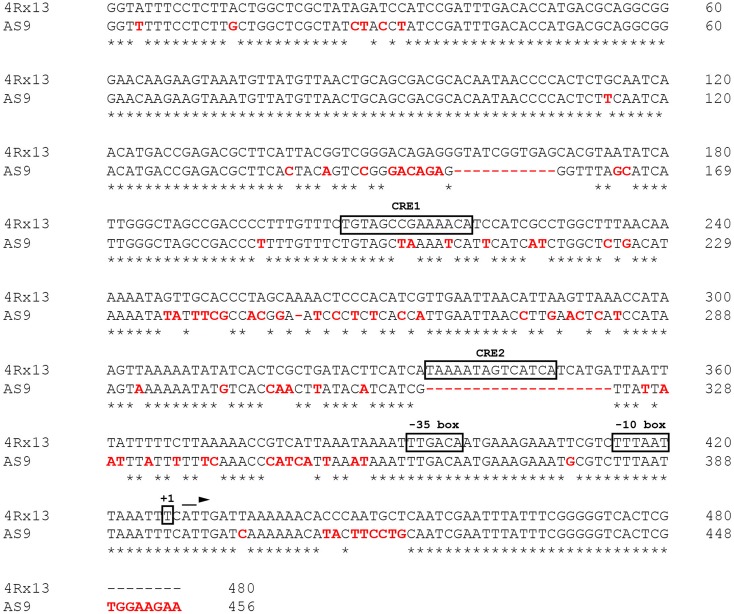
Analysis of the 5′-UTR in the sodorifen producer *Serratia plymuthica* 4Rx13 vs. the non-producing isolate *S.p.* AS9. Alignment was performed with the Clustal Omega online software (Sievers et al., 2011). Red letters indicate differences in *S.p.* AS9 in comparison to *S.p.* 4Rx13. Asterisks represent matches between both sequences and dashes deletions. Boxes highlight potential promoter sequences (–10/–35 box) and identified cAMP/CRP binding sites (CRE1/2) using the Regulatory Sequence Analysis Tool (RSAT, Medina-Rivera et al., 2015). +1 indicates transcription initiation point as determined by 5′-RACE PCR.

The differential expression of the sodorifen cluster genes was taken to indicate that regulation occurs at the transcriptional level. Subsequently, the nucleotide sequence directly upstream of the sodorifen cluster in *S.p.* 4Rx13 was compared with the respective sequences of the other producer strains *S.p.* HRO-C48, *S.p.* 3Re4-18, *S.p.* S13 and *S.p.* V4, as well as with the non-producer *S.p.* AS9 (**Figure 2** and Supplementary Figure S2, respectively). The sodorifen cluster upstream sequence was defined as the total number of nucleotides present between the first gene of the sodorifen cluster (in *S.p.* 4Rx13 = SOD_c20780; IPP isomerase) and the last gene upstream of the cluster (in *S.p.* 4Rx13 = SOD_c20790; putative oxidoreductase ydgJ). In *S.p.* 4Rx13, this sequence was 480 bp in length. In the other sodorifen producer strains the lengths were all similar, with 488 bp in *S.p.* HRO-C48 and *S.p.* S13, 479 bp in *S.p.* 3Re4-18 and 480 bp in *S.p.* V4. Contrary to this, the upstream sequence in the sodorifen non-producer *S.p.* AS9 was only 456 bp in length (**Figure 2**). Whereas among the sodorifen producers mainly substitutions and small insertions are apparent, two large deletions were found in *S.p.* AS9 ranging from nucleotides 158–169 and 335–355. Additionally, about 17% of the *S.p.* 4Rx13 sodorifen cluster upstream sequence was altered in *S.p.* AS9 due to nucleotide substitutions and therefore the total sequence identity (74.79%) was the lowest in comparison to the other sodorifen producer strains (94.38 to 98%). It became apparent that the sodorifen producers differ mostly at the same positions from the *S.p.* 4Rx13 sequence (Supplementary Figure S2). Furthermore, using 5′-RACE PCR it was found that the transcription of the sodorifen cluster initiates at a thymine nucleotide 53 bp upstream of the IPP isomerase start codon, making the identification of a putative promoter region (-10- and -35-box) and CRE 1 and 2 possible (**Figure 2**).

### Regulation of Sodorifen Emission by Carbon Catabolite Repression

**FIGURE 3:**
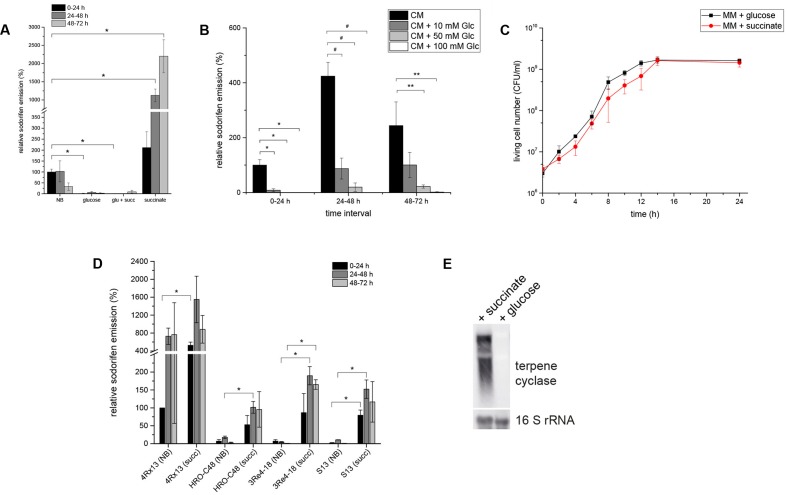
Regulation of the sodorifen emission in *Serratia* spp. by the carbon source. *S. plymuthica* 4Rx13 was cultivated in minimal medium supplemented with either 55 mM succinate, glucose, or a mixture of both (each 55 mM). **(A)** Relative sodorifen emission of *S.p.* 4Rx13. The volatiles were collected every 24 h by solid phase micro extraction (SPME) and analyzed using GC/MS. The peak area of sodorifen after 24 h cultivation in complex medium was used as reference (100%). ^∗^*p* ≤ 0.01. **(B)** Concentration dependent inhibition of the sodorifen emission in *S.p.* 4Rx13. ^∗^*p* ≤ 0.01; ^#^*p* < 0.005; ^∗∗^*p* < 0.05. **(C)** Growth of *S.p.* 4Rx13 in MM + 55 mM glucose and MM + 55 mM succinate. **(D)** Positive effects of succinate on the sodorifen emission in different sodorifen producing strains (*S.p.* 4Rx13, *S.p.* HRO- C48, *S.p.* 3Re4-18, *S.p.* S13). NB = complex medium (nutrient broth); succ = minimal medium + 55 mM succinate. For **(B,D)** sodorifen emission was assessed using the closed VOC collection system (modified after Kai et al., 2010) and analyzed by GC/MS. Quantification was performed with an internal standard (nonylacetate, 5 ng) and nutrient broth (NB) used as a reference. ^∗^*p* ≤ 0.05. **(E)** Expression level of the terpene cyclase gene of the sodorifen biosynthesis cluster in *S.p.* 4Rx13 after 24 h cultivation in MM + 55 mM succinate. Each lane contained 5 μg of total RNA. A DIG – dUTP – labeled probe was used for detection of terpene cyclase mRNA (upper panel). A second hybridization with a 16S rRNA probe was used as a positive control (lower panel). Hybridization of probes to the corresponding mRNAs was detected by fluorescence measurements for 1 min. Experiments were performed in triplicates and error bars represent standard deviation.

Sodorifen emission and gene expression was investigated during cultivation of *S.p*. 4Rx13 in various carbon sources. It was remarkable that sodorifen emission was about 20-fold higher during cultivation in minimal medium supplemented with 55 mM succinate than in complex medium (NB) (**Figure 3A**). Contrary to this, the relative sodorifen emission decreased to almost zero when glucose was added as the sole carbon source (MM + glucose), although the growth was not altered under the different nutrient conditions (**Figures 3A,C**, respectively). Furthermore, a concentration dependent inhibition of the sodorifen emission by glucose was observed in *S.p.* 4Rx13 ranging from 59% (10 mM glucose) to 98% (100 mM glucose) (**Figure 3B**), and simultaneous application of glucose and succinate (each 55 mM) caused reduced sodorifen emission compared to growth solely on succinate (**Figure 3A**).

The effect of succinate and glucose on the sodorifen emission in the other sodorifen producer strains *S.p.* HRO-C48, *S.p.* 3Re4-18, *S.p.* S13, and *S.p.* V4 was also assessed (**Figure 3D**). As a result, glucose inhibited the sodorifen emission completely (results not shown), whereas succinate, compared to complex medium, showed a stimulating effect on the sodorifen emission in these strains similar to *S.p.* 4Rx13. Surprisingly, the increase in the sodorifen emission was even more pronounced in the other producer strains, ranging from a 5.8-fold in *S.p.* HRO-C48 (24–48 h) to an over 200-fold increase in *S.p.* 3Re4-18 (48–72 h). The only exception was observed for the sodorifen producing strain *S.p.* V4, which was not capable of growing in MM + succinate for longer than 24 h, whereas growth in glucose-containing medium was comparable to *S.p.* 4Rx13 (results not shown). To assess a possible correlation between the sodorifen emission and the expression of the sodorifen biosynthesis genes, RT-PCR (Supplementary Figure S3) and Northern blots (**Figure 3E**) were performed. Quantification revealed that the transcription level of the terpene cyclase in MM + glucose is barely detectable and amounts to 5% compared to MM + succinate. The results obtained were further substantiated by experiments where either glucose was added to minimal medium + succinate during the stationary phase or vice versa (late addition of succinate to MM + glucose) (Weise et al., unpublished data). As expected, addition of glucose lead to a rapid decrease of sodorifen emission in succinate-containing medium, whereas addition of succinate did not further induce sodorifen production in MM + glucose. In conclusion, sodorifen emission is significantly reduced under glucose-containing conditions, whereas growth on succinate as the sole carbon source lead to a marked increase. These differences can be attributed to variations at the transcription level of the sodorifen cluster genes and are also observed in the other sodorifen producer strains. In all cases clear sodorifen production was only detectable in the absence of glucose, leading to the hypothesis that expression of the sodorifen biosynthesis genes is under control of CCR. This mode of regulation is well-known, and here it could be shown that CCR is implicated in the regulation of the biosynthesis of a volatile compound.

**FIGURE 4:**
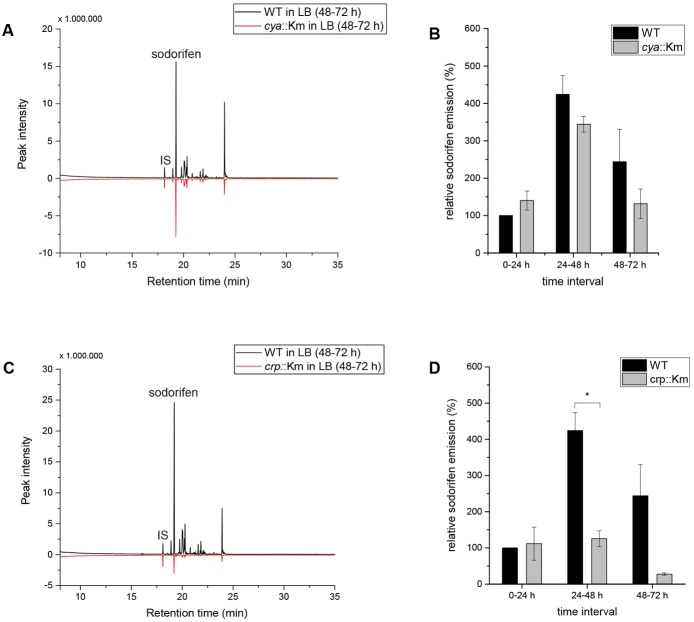
Sodorifen emission in *Serratia plymuthica* 4Rx13 and adenylate cyclase mutant (*cya*::Km) and cAMP receptor protein mutant (*crp*::Km). **(A,C)** Sodorifen emission of *cya*::Km/*crp*::Km mutants after 72 h cultivation in complex medium in comparison to the wild type of *S.p.* 4Rx13. **(B,D)** Relative sodorifen emission of *cya*::Km/*crp*::Km mutants during cultivation in complex medium. Sodorifen emission was determined using the closed VOC collection system (modified after Kai et al., 2010). For calculation of the relative sodorifen emission, the living cell number (CFU/ml) was correlated to the sodorifen amount measured. As a reference, wild type emission until 24 h cultivation was used (100%). Error bars indicate standard deviation (*n* = 3). ^∗^*p* < 0.01. IS, internal standard (nonylacetate, 5 ng).

This accumulating evidence for regulation of the sodorifen emission by CCR made the investigation of central CCR genes necessary. Different CCR regulation principles are known, e.g., in many Gram-negative bacteria CCR includes activation by a complex of cAMP and the cAMP receptor protein (Ruiz et al., 2010). The genes coding for the adenylate cyclase (cya) and for the cAMP receptor protein (crp) are central components of the CCR regulatory circuit and were selected for insertional deletion mutagenesis in *S.p.* 4Rx13. Integration of a functional cassette coding for a kanamycin resistance gene was performed by homologous recombination. Unhindered growth and the correct insertion and stability of the functional cassette in the mutants were verified by polymerase chain reaction (Supplementary Figures S4A–F). The results indicate that the insertion in the *cya* or the *crp* gene in *S.p.* 4Rx13 did not affect the growth of the mutants in comparison to the wild type (Supplementary Figures S4B,E), while comparison of the VOC profiles of both, wild type and *cya*::Km revealed a reduction in the sodorifen emission in the mutant of up to 50% (**Figure 4A**) and for the *crp*::Km mutant an almost 10-fold reduction of the sodorifen emission was observed (**Figure 4C**). To ensure comparability between the wild type and the mutants, the relative sodorifen emission was calculated by relating the living cell number of each culture to the peak intensity of sodorifen measured by GC/MS. The results revealed that, again, in the wild type, as well as in the mutants, the sodorifen emission increased until 48 h of growth in complex medium, and thereafter decreased (**Figures 4B,D**). Direct comparison of the wild type emission levels to those reached by the mutants showed that in the time interval of 0–24 h the relative sodorifen emission of the *cya* mutant exceeded that of the wild type by about 40% (**Figure 4B**). In the time intervals 24–48 and 48–72 h the sodorifen emission in the mutants was less compared to the wild type; pronounced lower levels were observed for the *crp* mutant (**Figure 4D**).

Since for both mutants, *cya*::Km and *crp*::Km, an overall negative effect on the emission of sodorifen was measured, it was speculated whether this effect correlated with a decrease of the sodorifen cluster gene expression. Since quantification of the sodorifen cluster expression is barely detectable after 48 h/72 h cultivation (Supplementary Figure S1B), only expression levels after 24 h of growth were assessed (Supplementary Figure S4G). As expected, quantification indicated that the expression of the terpene cyclase after 24 h cultivation in complex medium was lowest in *S.p.* 4Rx13 WT (100%), while in the *crp* and the *cya* insertion mutants the expression levels were ca. 3–4 times higher than in the wild type strain (321 and 380%, respectively) reflecting the increased sodorifen emission in these mutants during the time interval 0–24 h (**Figures 4B,D**).

**FIGURE 5:**
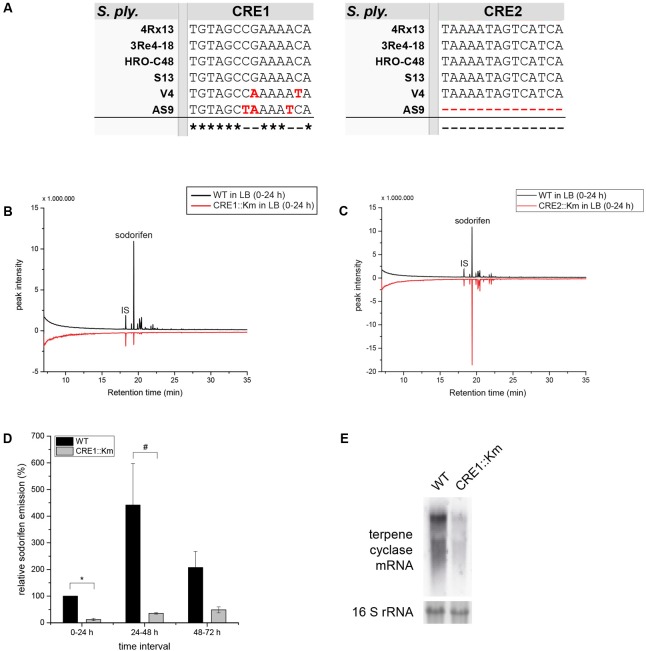
Characterization of CRE1::Km and CRE2::Km deletion mutants. **(A)** Alignment of the two potential CRE binding sites in the sodorifen cluster upstream sequence from *S.p.* 4Rx13. Alignment was performed using the Clustal Omega online software (Sievers et al., 2011). Black letters represent nucleotides matching the sequence of *S.p.* 4Rx13, red letters indicate differences. Asterisks mark identities between all strains tested, dashes represent alterations. **(B,C)** Sodorifen emission of CRE1::Km/CRE2::Km deletion strains after 24 h cultivation in minimal medium + 55 mM succinate in comparison to the wild type of *S.p.* 4Rx13. **(D)** Relative sodorifen emission of CRE1::Km during cultivation in MM + succinate. For calculation of the relative sodorifen emission, the living cell number (CFU/ml) was correlated to the sodorifen amount measured. As a reference, wild type emission until 24 h cultivation was used (100%). Error bars indicate standard deviation (*n* = 3). ^∗^*p* < 0.01; ^#^*p* < 0.05. Sodorifen emission in **(B–D)** was determined using the closed VOC collection system (modified after Kai et al., 2010) with subsequent GC/MS analysis. IS, internal standard (nonylacetate, 5 ng). **(E)** Expression of the terpene cyclase in *S.p.* 4Rx13 wild type and CRE1::Km. Northern blot was performed with 5 μg RNA isolated after 24 h cultivation in MM + 55 mM succinate. Detection of 16S rRNA expression level was used as a positive control and to ensure equal RNA loading. Expression was detected using DIG – dUTP – labeled probes and fluorescence measurement s for 1 min.

Other important key players in CCR are the binding sites upstream from the promoter, which facilitate the binding of RNA polymerase and subsequently transcription. These sequences are referred to as CRE sites. It is known that a complex built of cAMP and its receptor protein (CRP) binds to these recognition sequences. By using a consensus sequence for CRE sites (Kant et al., 2009), the 5′ untranslated region (5′-UTR) of the sodorifen cluster was screened and two possible binding sites of the cAMP/CRP complex (CRE1/2) with an identity of 85.7 and 78.6%, respectively, to the consensus sequence were found (**Figure 2**). Furthermore, the potential CRE sites identified in *S.p.* 4Rx13 were searched for in other sodorifen producer (*S.p.* HRO-C48, *S.p.* 3Re4-18, *S.p.* S13, *S.p.* V4) and one non-producer strain (*S.p.* AS9) and their sequences were aligned (**Figure 5A**). The results clearly showed that the sodorifen producer strains *S.p.* 3Re4-18, *S.p.* HRO-C48, and *S.p.* S13 possess potential CRE sites, which are 100% identical to those in *S.p.* 4Rx13. The only exception was the sodorifen emitting isolate *S.p.* V4, where the CRE1 sequence differs in two positions from the one in *S.p.* 4Rx13, whereas CRE2 is again identical. However, more sequence variations can be found when comparing the sodorifen producing isolate *S.p.* 4Rx13 with the non-producer *S.p.* AS9. In the case of CRE1 the identity is 78.6% whereas CRE2 was completely missing in *S.p.* AS9. The effect of the identified cAMP/CRP binding sequences, CRE1 and CRE2, on the sodorifen emission was investigated by constructing deletion mutants where the respective 14 bp sequences were removed and replaced by a kanamycin resistance gene (Supplementary Figure S5). For CRE2::Km it became apparent that no differences in sodorifen emission occurred in comparison to the wild type strain of *S.p.* 4Rx13 regardless whether the bacteria grow on minimal medium plus succinate (**Figure 5C**) or on LB medium (Supplementary Figure S6). In contrary, the CRE1 deletion strain showed a strong reduction in the emission of sodorifen and all of its potential derivatives (isomers) (in minimal medium plus succinate: **Figure 5B**, in complex medium: Supplementary Figure S6). Apart from this, no qualitative variations were detectable in the VOC profile. Quantitative analysis of the sodorifen emission of CRE1::Km in comparison to *S.p.* 4Rx13 WT was performed in minimal medium supplemented with succinate by relating the amount of emitted sodorifen to the number of viable cells (**Figure 5D**). In the wild type, sodorifen emission gradually increased from 100% up to ca. 270% after 72 h cultivation. For the CRE1 mutant a constant increase in sodorifen emission was observed over time and in contrast to the WT, the measured sodorifen amounts only reached values ranging from 12 to 48% representing a reduction in sodorifen emission of 82–88% in CRE1::Km mutant in comparison to the WT, respectively.

Furthermore, the influence of the CRE1 deletion on the sodorifen cluster expression was assayed. Exemplarily, the mRNA level of the terpene cyclase was investigated by Northern blot after 24 h cultivation of the wild type and the CRE1 mutant (**Figure 5E**) in minimal medium containing succinate as the sole carbon source. Quantification revealed an expression level of 21% for the terpene cyclase in this mutant in comparison to the wild type (100%).

In conclusion, deletion of the potential cAMP/CRP binding site CRE2 from the sodorifen cluster 5′-UTR had no effect on the sodorifen emission, whereas deletion of CRE1 lead to a significant decrease of up to 88% in sodorifen emission in comparison to the wild type. Moreover, the expression level of the terpene cyclase gene was reduced by about 79% in the CRE1 deletion strain, indicating a direct regulation of the sodorifen biosynthesis by CCR.

## Discussion

The initial observation that the emission profile of small VOCs was significantly influenced when *S. plymuthica* 4Rx13 was grown on different media was further elaborated. Here, we provide evidence that the emission of the unique VOC sodorifen is tightly regulated at the transcriptional level, supported by the fact that it is a matter of induction of the sodorifen biosynthesis cluster which is not expressed in the genetically close relative *S.p.* AS9, which is thus not capable of producing sodorifen (Domik et al., 2016a,b). Moreover, the novel hypothesis of CCR acting as a major control entity on the synthesis of mVOCs was proven by genetic evidence. Central components of the typical CCR of Gram-negative bacteria such as the adenylate cyclase (CYA), the cAMP binding receptor protein (CRP), and the CRE binding sites were removed by insertional deletion and mutants revealed the expected lower sodorifen emission.

### Sodorifen Emission Is Regulated at the Transcriptional Level

The related *S. plymuthica* strains investigated in our research shared the four genes present in the sodorifen cluster, with 95–100% sequence identity (Domik et al., 2016a,b). Here, we present evidence that the sodorifen cluster expression is not only under qualitative regulation (expression on/off) but is also quantitatively controlled. According to the lower sodorifen cluster expression status in *S.p.* HRO-C48, *S.p.* S13 and *S.p.* V4, also low emission of sodorifen was observed. Surprisingly, in *S.p.* 3Re4-18 the expression level of the sodorifen cluster was found to be even higher than in *S.p.* 4Rx13, although the emission levels were more than 10-fold lower. Despite high transcription of the sodorifen cluster, we hypothesize that either the translation in *S.p.* 3Re4-18 is impaired or the resulting proteins are less efficient. In our previous work we could show that the sodorifen producing strains *S.p.* HRO-C48 and *S.p.* V4 possess four amino acid exchanges in their terpene cyclase proteins (Domik et al., 2016b). These variations are also present in *S.p.* 3Re4-18 and might be the reason for its low sodorifen emission which was comparable to *S.p.* HRO-C48. Nevertheless, sodorifen cluster expression in the producer strain *S.p.* 3Re4-18 must be differently regulated compared to *S.p.* 4Rx13. To assess this hypothesis, the 5′-UTR of the sodorifen cluster was analyzed in all known sodorifen producers as well as in the non-producer *S.p.* AS9 (Supplementary Figure S2). Very high homology of the nucleotide sequence upstream of the sodorifen cluster among all producer strains (94–98%) were observed; only the non-producer *S.p.* AS9 showed several deviations. Subsequently, it is postulated that trans-elements (e.g., transcription factors and miRNAs) might be responsible for the elevated expression levels in *S.p* 3Re4-18. This hypothesis remains to be investigated. In turn, results of non-expression of the sodorifen cluster genes in *S.p.* AS9 might be due to sequence alterations and deletions that lead to an inability of potential transcription factors to bind to the 5′-UTR and subsequently to the sodorifen-negative phenotype of *S.p.* AS9. It is further remarkable that the sodorifen-producers *S.p.* 4Rx13, *S.p.* HRO-C48, *S.p.* 3Re4-18, *S.p.* S13, and *S.p.* V4 all shared a very high identity of their upstream sequences but still the latter four exhibited only low sodorifen production. One explanation can be found at the end of the 5′-UTR which is either elongated by eight nucleotides (in *S.p.* HRO-C48 and *S.p.* S13) or a guanine was eliminated (*S.p.* 3Re4-18 and *S.p.* V4) right before the translational start-codon of the first gene in the sodorifen cluster. These deviations would lead to a shift of the potential promoter sequence and therefore might influence the transcription efficiency of the sodorifen cluster genes. Another explanation would be that additional transcription factors are involved in induction of the sodorifen cluster expression whose effects are less pronounced in the producer strains, other than *S.p.* 4Rx13, because of the overall differences in the nucleotide upstream sequence. Identification of potential transcription factors acting on the sodorifen cluster expression are subject of current investigations.

Apart from the differential regulation among the sodorifen producing isolates, shared regulatory mechanisms became apparent. For example, feeding different carbon sources to *S.p.* 4Rx13 revealed a strong, concentration dependent inhibitory effect of glucose on the sodorifen emission, whereas succinate significantly increased sodorifen production without altering the growth of the bacterium (**Figures 3A–C**). These effects could be attributed to a lower or higher expression of the sodorifen cluster genes in *S.p.* 4Rx13 during cultivation in the respective media. The same trend in sodorifen emission upon growth on glucose and succinate was observed in the producers *S.p.* HRO-C48, *S.p.* 3Re4-18, *S.p.* S13 and *S.p.* V4 (**Figure 3D**), indicating that in this regard conserved regulatory mechanisms are manifested.

### Sodorifen Emission and Carbon Catabolite Repression

**FIGURE 6:**
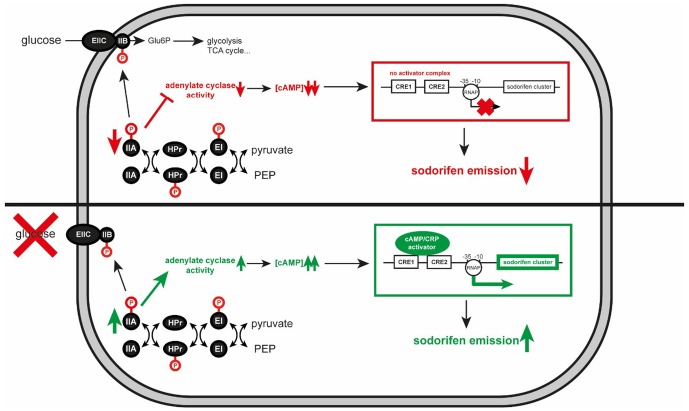
Schematic model of the influence of carbon catabolite repression (CCR) on the sodorifen emission in *Serratia plymuthica* 4Rx13. Red and green arrows indicate decrease and increase, respectively. Blocked lines represent inhibitory effect. –10 and –35 indicate the promoter of the sodorifen cluster. Glu6P, glucose-6-phosphate; TCA, tricarboxylic acid cycle; P, phosphate group; PEP, phosphoenolpyruvate; RNAP, RNA polymerase; EI, HPr, IIA, components of the phospho transferase system; EIIC and IIB, glucose transporting system; CRE, carbon catabolite responsive element; CRP, cAMP receptor protein.

Particularly interesting was the observation that addition of glucose to the medium dramatically reduced the emission of sodorifen, a phenomenon that was – to the best of our knowledge – so far not studied in the context of mVOC emission elsewhere. Nevertheless, the effect of the carbon source on secondary metabolite formation has been the subject of many studies, both from industry and research groups. Following the dictum of Demain (1989) ‘too much of a good thing can be bad,’ glucose turned out to be the preferred carbon source in a mixture of rapidly and slowly-used carbon sources, however, with the drawback of little or no synthesis of secondary metabolites. Several mechanisms have been described in bacteria and fungi to explain the negative carbon catabolite effects on secondary metabolite production (Gallo and Katz, 1972; Aharonowitz and Demain, 1978; Kwakman and Postma, 1994; van Wezel and McDowall, 2011; Huang et al., 2012; Liu et al., 2013). Due to extensive antibiotic production in Actinomycetales (e.g., *Streptomyces*) the underlying regulatory networks and mechanisms involved in the biosynthesis of these secondary metabolites were well-studied in these and other Gram-positive bacteria, and often a PTS depending system was demonstrated to be involved (summarized in Ruiz et al., 2010). In Gram-negative organisms, it was speculated whether the PTS depending system is also active by hindering other carbon sources to enter the cell (PTS-mediated inducer exclusion; Lengeler and Postma, 1998). Based on the *E. coli* CCR system we constructed a model for the regulation of the sodorifen synthesis by CCR in *S. plymuthica* (**Figure 6**). This homologous model suggests that inactivation of CCR would diminish the synthesis of secondary metabolites, in the case of *S. plymuthica* specifically the sodorifen emission. The *E. coli* model comprises three high-energy phosphoprotein intermediates and five protein domains. EIIA is phosphorylated by the phosphoprotein HPr, and transfers the phosphate to EIIB/C that resides at the cell membrane as a homodimer and then further to glucose, thus rendering glucose-6-phosphate. Consequently, in the presence of glucose, EIIA is predominantly found in its dephosphorylated form, resulting in inactivation of transporters for other sugar sources and furthermore in low adenylate cyclase activity (encoded by the *cya* gene). Since adenylate cyclase produces cAMP, high glucose levels result in low cAMP concentrations. Absence of cAMP, in turn, impedes complex formation with the cAMP receptor protein, which leads to non-activation of target operon expression. In contrast to this, low glucose levels result in accumulation of phosphorylated EIIA∼P, which activates adenylate cyclase. Consequently, cAMP levels increase and formation of the cAMP/CRP complex takes place. This activator complex can now bind to CRE sequences upstream of target operons and enhance its expression. To determine the role of CCR in the regulation of sodorifen biosynthesis, insertional mutagenesis was performed on the central CCR genes: *cya* coding for the adenylate cyclase, which is responsible for production of the second messenger cAMP, and *crp* representing the cAMP receptor protein. Both mutants showed – as expected – an overall reduced sodorifen emission compared to the wild type, already indicating an involvement of CCR in the regulation of sodorifen emission. What is surprising is the fact that the *cya* mutant strain exhibited much higher sodorifen emissions than *crp*::Km mutant. The reason for this rather contradictory result is still not entirely clear. The possibility that additional *cya* genes might be present in the genome of *S.p.* 4Rx13 was ruled out since no homologous or annotated gene was detected. Another assumption would be that nucleotides other than cAMP could also bind to CRP leading to the formation of analogous complexes, which in turn can bind less efficiently to the sodorifen cluster upstream sequence. So far, several studies could show that variations in the amino acid sequence of CRP lead to either variations in ligand specificity or even to constitutively active CRP proteins (Garges and Adhya, 1985; Ryu et al., 1993; Lee et al., 1994; Youn et al., 2006). Comparison of the amino acid sequences of the CRP protein in *E. coli* K-12 with *S.p.* 4Rx13 revealed an identity of 99.52% with a R123S substitution. So far, nothing is known about the effect of such an alteration in the CRP protein and, to this point, it can only be speculated that it could lead to alteration in the CRP activity in *S.p.* 4Rx13. Consequently, sodorifen emission would be observable in the *cya*::Km mutant in larger quantities than in the *crp*::Km mutant, where binding to the 5′-UTR of the sodorifen cluster no longer takes place. Furthermore, Li et al. (2008) reported the effect of ATP on the antibiotic formation in *Streptomyces coelicolor*. When this microorganism is grown in the presence of 10 mM ATP, the actinorhodin concentration increased by 90% compared to a culture grown in the absence of the nucleotide.

Another surprising result was the high sodorifen emission and especially cluster expression in *cya*::Km and *crp*::Km mutants after 24 h cultivation in complex medium, which even exceeded that of the wild type. However, Wang et al. (2005) could show that the *lux*S gene, which is responsible for production of the quorum sensing signal autoinducer-2, is under negative control by cAMP/CRP. In an additional experiment we saw that upon inactivation of *cya* and *crp* indeed the expression level of *lux*S was increased (Magnus and Piechulla, unpublished). Therefore, quorum sensing mechanisms could lead to induction of sodorifen cluster expression independent of CCR.

To further support the idea that the sodorifen cluster expression is regulated by CCR, two potential binding sites for the cAMP/CRP complex (CRE1/2) were identified in the 5′-UTR of the cluster and eliminated. CRE motifs have often been shown to be essential for binding of the cAMP/CRP complex (e.g., Villarreal et al., 2011; Herrera et al., 2012; Aung et al., 2014) using electrophoretic mobility shift assays (EMSA) and therefore provide evidence for a direct influence of CCR on operon expression. It is reasonable to hypothesize that binding of cAMP/CRP only takes place at CRE1 upstream of the sodorifen cluster, because of its higher identity to the consensus sequence and thus, higher affinity to the complex, but especially because of its positive effect on the sodorifen production. Nevertheless, simultaneous binding of two cAMP/CRP complexes, to CRE1 and CRE2, respectively, cannot be excluded. For example, it was shown for the *E. coli* deoP_2_ promoter that binding of two cAMP/CRP complexes to two distinct CRE sites is necessary for the cytidine repressor (CytR) to bind and suppress expression of catabolic genes for nucleosides (Valentin-Hansen et al., 1996; Chahla et al., 2003). In this case, the two CRE sites exhibit different binding efficiencies for the cAMP/CRP complex with the most upstream one having the highest affinity. Binding of one or two cAMP/CRP complexes enhances expression of the following operon, whereas occupation of both CRE sites also renders the opportunity for the CytR repressor to bind and stop expression. A similar system might be active in case of the sodorifen cluster. Consequently, CRE1 would act as binding site for enhancing sodorifen cluster expression, whereas occupancy of CRE2 would lead to recruitment of an additional repressor. As a result, deletion of CRE2 would have no repressing effect on the sodorifen emission, in contrast to CRE1. To test this hypothesis, identification of possible repressors is essential and it will be necessary to prove cAMP/CRP binding to the potential CRE sites. Moreover, the importance of CRE2 and/or 1 in sodorifen emission was underpinned by the sodorifen-negative strain *S.p.* AS9, where three nucleotide exchanges were present in CRE1, and CRE2 was completely missing. It will be of great interest in the future to exchange the *S.p.* AS9 5′-UTR, in part or completely, with the upstream sequence from *S.p.* 4Rx13 to see if only the lack of binding sites for transcriptional inducers, e.g., cAMP/CRP, is responsible for its non-sodorifen-producing phenotype. Nevertheless, the negative effect of CRE1 deletion on the sodorifen emission in *S.p.* 4Rx13 clearly showed that CCR is directly involved in regulation of the sodorifen biosynthesis.

Our work provides evidence that sodorifen production is under tight transcriptional control and to our knowledge, it was demonstrated for the first time that bacterial VOC emission is regulated by CCR. This is remarkable, because presently only a small number of examples are known where secondary metabolites are suppressed in the presence of specific carbon sources, while CCR is a well-known concept to control primary metabolism (summarized in Ruiz et al., 2010). For instance, glucose depresses formation of aminoglycoside antibiotic via repression of the biosynthetic enzymes (Demain, 1989; Piepersberg and Distler, 1997). Also, production of β-lactam antibiotics and macrocyclic polyketides are regulated by the carbon source. Often, respective repressions are manifested at the transcriptional level, and a similar regulation concept was found for the sodorifen cluster genes of *S. plymuthica*. The knowledge about regulatory cascades and networks that govern or operate secondary metabolism is particularly valuable when respective compounds such as antibiotics are considered for applications or have economic impact. mVOCs also match these criteria because quite a large number of these compounds are part of important aroma bouquets in foodstuffs, e.g., cheese, wine, beer, yogurt, and were selected for human preferences. Furthermore, attention was given to mVOCs which function as indicators for contaminations and pollutants with potential consequences for human health (Korpi et al., 2009), or those which will be used as diverse medical tools, e.g., specific diagnosis approaches, or are part of new approaches in agriculture and biotechnology (summarized in Piechulla and Lemfack, 2016). Finally, secondary metabolites of novel and unusual structures may be used as lead structures for new bioactive compounds.

## Author Contributions

NM designed and performed all experiments, except those for **Figures 3A,B** which were done by TW. BP supervised the experiments. The manuscript was written by NM and BP.

## Conflict of Interest Statement

The authors declare that the research was conducted in the absence of any commercial or financial relationships that could be construed as a potential conflict of interest.
