# Supplementary material for: Carbon Catabolite Repression Regulates the Production of the Unique Volatile Sodorifen of Serratia plymuthica 4Rx13
Source: Front Microbiol. 2017 Dec 19;8:2522. doi: 10.3389/fmicb.2017.02522 (PMC5742105; doi:10.3389/fmicb.2017.02522)
Supplement: Supplementary file 1 [file Data_Sheet_1.pdf]

Journal name:

Frontiers in Microbiology

Manuscript title:

Carbon catabolite repression regulates the production of the unique volatile sodorifen of *Serratia plymuthica* 4Rx13

Authors:

Nancy Magnus[1], Teresa Weise[2], Birgit Piechulla[1]*

present addresses:

1University of Rostock, Institute for Biological Sciences, Albert-Einstein-Str. 3, 18059 Rostock, Germany

2 EuroImmun, Medizinische Labordiagnostik AG, Seekamp 31, 23560 Lübeck

***corresponding author**

e-mail: birgit.piechulla@uni-rostock.de;

phone: 0049 381 4986130

fax: 0049 381 4986132

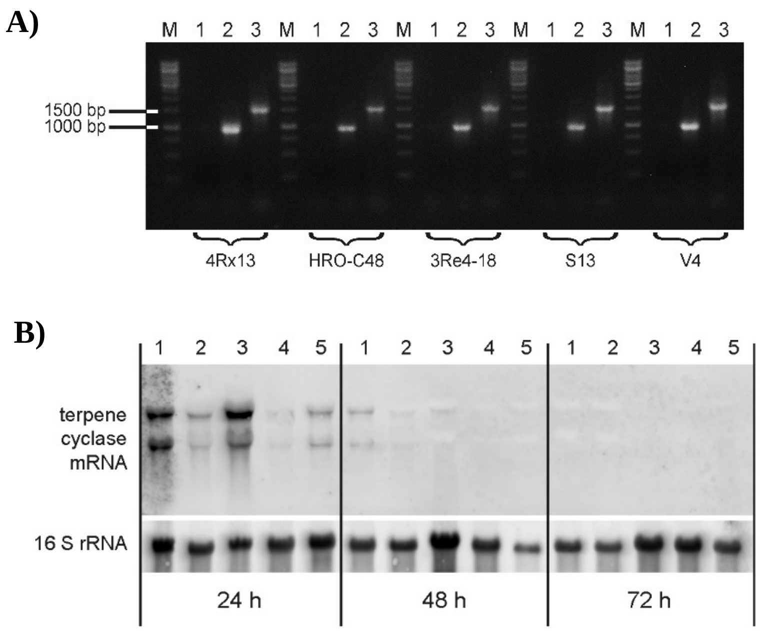

Figure S1: Expression of sodorifen cluster in different producer strains. Expression status of the sodorifen cluster genes was assessed in the different producer strains *Serratia plymuthica* 4Rx13, HRO-C48, 3Re4-18, S13 and V4 after 24 h cultivation in minimal medium + 55mM succinate. **A)** RT-PCR was performed with primers specific for the terpene cyclase gene of the sodorifen cluster to prove expression in all producing strains (lane 2). Lane 1: negative control (without reverse transcriptase), lane 3: positive control (16 S rRNA). For each bacterial isolate 1 µg of RNA was used as a template. M = marker (GeneRuler 1 kb). **B)** Northern blot was performed to quantify expression levels of the terpene cyclase gene in the sodorifen producers after 24 h, 48 h and 72 h cultivation (1: *S.p.* 4Rx13, 2: *S.p.* HRO C48, 3: *S.p.* 3Re4-18, *S.p.* S13, 5: *S.p.* V4). Blotting was done with 5 µg RNA each and DIG-dUTP-labelled probes specific for the terpene cyclase (upper panel) and the 16 S rRNA (lower panel) as a positive control. Finally, labelled RNA molecules were detected by fluorescence measurement for 1 min.

```
4Rx13       GGTATTTCCTCTTACTGGCTCGCTATAGATCCATCCGATTTGACACCATGACGCAGGCGG   60
HRO-C48     GGTATTTCCTCTTGCTGGCTCGCTATAGATCCATCCGATTTGACACCATGACGCAGGCGG   60
3Re4-18     GGTATTTCCTCTTGCTGGCTCGCTATAGATCCATCCGATTTGACACCATGACGCAGGCGG   60
S13         GGTATTTCCTCTTGCTGGCTCGCTATAGATCCATCCGATTTGACACCATGACGCAGGCGG   60
V4          GGTATTTCCTCTTGCTGGCTCGCTATAGACCCATCCGATTTGACACCATGACGCAGGCGG   60
            ************* *************** ******************************

4Rx13       GAACAAGAAGTAAATGTTATGTTAACTGCAGCGACGCACAATAACCCCACTCTGCAATCA   120
HRO-C48     GAACAAGAAGTAAATGTTATGTTAACTGCAGCGACGCACAATAACCCCACTCTGCAATCA   120
3Re4-18     GAACAAGAAGTAAATGTTATGTTAACTGCAGCGACGCACAATAACCCCACTCTGCAATCA   120
S13         GAACAAGAAGTAAATGTTATGTTAACTGCAGCGACGCACAATAACCCCACTCTGCAATCA   120
V4          GAACAAGAAGTAAATGTTATGTTAACTGCAGCGACGCACAATAACCCCACACTGCAATCA   120
            ************************************************** *********

4Rx13       ACATGACCGAGACGCTTCATTACGGTCGG-GACAGAGGGTATCGGTGAGCACGTAATATC   179
HRO-C48     ACATGACCGAGACGCTTCATTACGGTCGG-GACAGAGGGTATCGGTGAGCACGTAACATC   179
3Re4-18     ACATGACCGAGACGCTTCATTACGGTCGG-GACAGAGGGTATCGGTGAGCACGTAACATC   179
S13         ACATGACCGAGACGCTTCATTACGGTCGG-GACAGAGGGTATCGGTGAGCACGTAACATC   179
V4          ACATGACCGAGACGCTTCATTACGGTCGGGGACAGAGGGTATCGGTGAGCACGTAACATC   180
            ***************************** ************************** ***

4Rx13       ATTGGGCTAGCCGACCCCTTTGTTTCTGTAGCCGAAAACATCCATCGCCTGGCTTTAACA   239
HRO-C48     ATTGGGCTAGCCGACCCTTTTGTTTCTGTAGCCGAAAACATCCATCGCCTGGCTTTAACA   239
3Re4-18     ATTGGGCTAGCCGACCCTTTTGTTTCTGTAGCCGAAAACATCCATCGCCTGGCTTTAACA   239
S13         ATTGGGCTAGCCGACCCTTTTGTTTCTGTAGCCGAAAACATCCATCGCCTGGCTTTAACA   239
V4          ATTGGACTAGCCGACCCTTTTGTTTCTGTAGCCAAAAATATTCATCACCTGGCTTTGACA   240
            ***** *********** *************** **** ** **** ********* ***

4Rx13       AAAAATAGTTGCACCCTAGCAAAACTCCCACATCGTTGAATTAACATTAAGTTAAACCAT   299
HRO-C48     AAAAATAGTTGCACCCTAGCAAAACTCCCACATCGTTGAATTAACATTAAGTTAAACCAT   299
3Re4-18     AAAAATAGTTGCACCCTAGCAAAACTCCCACATCGTTGAATTAACATTAAGTTAAACCAT   299
S13         AAAAATAGTTGCACCCTAGCAAAACTCCCACATCGTTGAATTAACATTAAGTTAAACCAT   299
V4          TAAAATAGTTTCGCCATAGCAAAACTCCCACATCGTTGAATTAACATTAAGTTAAACCAT   300
             ********* * ** ********************************************

4Rx13       AAGTTAAAAATATATCACTCGCTGATACTTCATCATAAAATAGTCATCATCATGATTAAT   359
HRO-C48     AAGTTAAAAATATATCACCAGCTGATACTTCATCATAAAATAGTCATCATCATGATTAAT   359
3Re4-18     AAGTTAAAAATATATCACCAGCTGATACTTCATCATAAAATAGTCATCATCATGATTAAT   359
S13         AAGTTAAAAATATATCACCAGCTGATACTTCATCATAAAATAGTCATCATCATGATTAAT   359
V4          AAGTTAAAAATATATCACCAGCTGATACTTCATCATAAAATAGTCATCATCATGATTAAT   360
            ******************  ****************************************

4Rx13       TTATTTTTCTTAAAAACCGTCATTAAATAAAATTTGACAATGAAAGAAATTCGTCTTTAA   419
HRO-C48     TTATTTTTATTAAAAGCCATCATTAAATAAAATTTGACAATGAAAGAAATGCGTCTTTAA   419
3Re4-18     TTATTTTTATTAAAAGCCATCATTAAATAAAATTTGACAATGAAAGAAATGCGTCTTTAA   419
S13         TTATTTTTATTAAAAGCCATCATTAAATAAAATTTGACAATGAAAGAAATGCGTCTTTAA   419
V4          TTATTTTTCTTAAAAACCATCATTAAATAAAGTTTGACAATGAAAAAAATGCGTCTTTAA   420
            ******** ****** ** ************ ************* **** *********

4Rx13       TTAAATTTCATTGATTAAAAAACACCCAATGCTCAATCGAATTTATTTCGGGGGTCACTC   479
HRO-C48     TTAAATTTCATTGATCAAAAAACACCCAATGCTCAATCGAATTTATTTCGGGGGTCACTC   479
3Re4-18     TTAAATTTCATTGATCAAAAAACACCCAATGCTCAATCGAATTTATTTCGGGGGTCACTC   479
S13         TTAAATTTCATTGATCAAAAAACACCCAATGCTCAATCGAATTTATTTCGGGGGTCACTC   479
V4          TTAAATTTCATTGATCAAAAAACACCCTATCTGCAATCGAATTTATTTCGGGGGTCACTC   480
            *************** *********** **   ***************************

4Rx13       G--------        480
HRO-C48     GTGGAAGAA        488
3Re4-18     ---------        479
S13         GTGGAAGAA        488
V4          ---------        480
```

Figure S2: Analysis of the 5'-UTR in the sodorifen producer strains *Serratia plymuthica.* 4Rx13, HRO-C48, 3Re4-18, S13 and V4. Alignment was performed with the Clustal Omega online software (Sievers et. al. 2011). Red letters indicate differences in the sodorifen producing strains in comparison to *S. p.* 4Rx13. Asterisks represent matches between all sequences and dashes deletions.

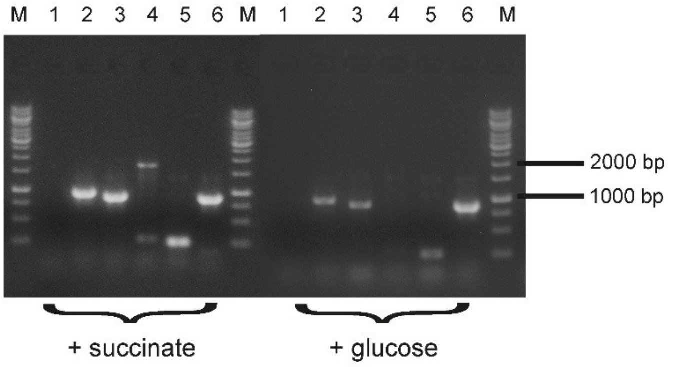

Figure S3: Expression of the sodorifen cluster depending on the carbon source. Expression status of the sodorifen cluster genes was assessed in *Serratia plymuthica* 4Rx13 after 24 h cultivation in minimal medium supplemented with either 55 mM succinate or glucose. RT-PCR was performed with primers specific for the terpene cyclase (lane 2), methyltransferase (lane 3), DOXP synthase (lane 4) and IPP isomerase (lane 5) of the sodorifen cluster. Lane 1: negative control (without reverse transcriptase), lane 6: positive control (GAPDH). For each cultivation condition, 1 µg of RNA was used as a template. M = marker (GeneRuler® 1kb).

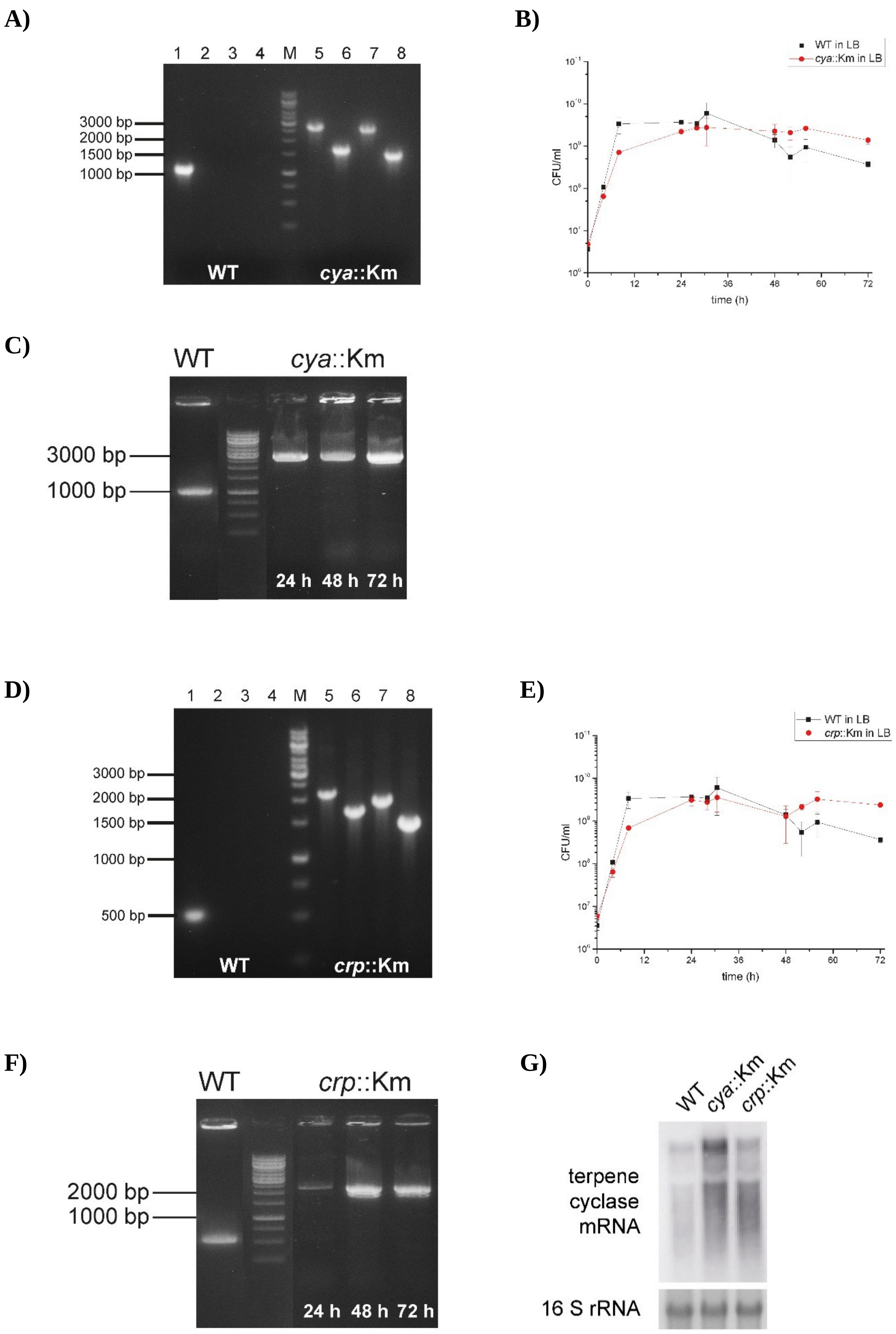
A)
1 2 3 4 M 5 6 7 8
3000 bp
2000 bp
1500 bp
1000 bp
WT
cya::Km
B)
WT in LB
cya::Km in LB
CFU/ml
time (h)
0 12 24 36 48 60 72
C)
WT
cya::Km
3000 bp
1000 bp
24 h 48 h 72 h
D)
1 2 3 4 M 5 6 7 8
3000 bp
2000 bp
1500 bp
1000 bp
500 bp
WT
crp::Km
E)
WT in LB
crp::Km in LB
CFU/ml
time (h)
0 12 24 36 48 60 72
F)
WT
crp::Km
2000 bp
1000 bp
24 h 48 h 72 h
G)
WT
cya::Km
crp::Km
terpene
cyclase
mRNA
16 S rRNA

Figure S4: Characterization of the *cya*::Km and *crp*::Km insertion mutants of *Serratia plymuthica* 4Rx13. A)-C) Characterization of *cya*::Km. **A)** Verification of correct insertion of the functional cassette by PCR. In lanes 1-4 results for the *S. p.* 4Rx13 wildtype are shown and in lanes 5-8 for the *cya* mutant. Lanes 1 & 5 represent amplification products with gene-specific primers for the *cya* gene (1.1 kb for the WT; 2.7 kb for *cya*::Km). Lanes 4 & 8 depict amplification products of primers specific for the functional cassette (1.4 kb). Lanes 2 & 6 show PCR products after amplification with gene- and cassette-specific primers (1.6 kb; left border), as well as lanes 3 & 7 (right border; 2.5 kb). M = marker (GeneRuler® 1 kb). **B)** Growth comparison of *S. p.* 4Rx13 WT and *cya*::Km in complex medium (NB). The living cell number (CFU/ml) was determined periodically for up to 72 h. Error bars represent standard deviation (n=3). **C)** Stability of the mutation of *cya* in *S. p.* 4Rx13 was determined in 24 h intervals for up to 72 h of cultivation in complex medium (LB) using PCR. **D)-F)** Characterization of *crp*::Km. **D)** Verification of correct insertion of the functional cassette by PCR. In lanes 1-4 results for the *S. p.* 4Rx13 wildtype are shown and in lanes 5-8 for the *crp* mutant. Lanes 1 & 5 represent amplification products with gene-specific primers for the *crp* gene (537 bp for the WT; 2.15 kb for *crp*::Km). Lanes 4 & 8 depict amplification products of primers specific for the functional cassette (1.4 kb). Lanes 2 & 6 show PCR products after amplification with gene- and cassette-specific primers (1.68 kb; left border), as well as lanes 3 & 7 (right border; 1.89 kb). M = marker (GeneRuler® 1 kb). **E)** Growth comparison of *S. p.* 4Rx13 WT and *crp*::Km in complex medium (NB). The living cell number (CFU/ml) was determined periodically for up to 72 h. Error bars represent standard deviation (n=3). **F)** Stability of the mutation of *crp* in *S. ply.* 4Rx13 was determined in 24 h intervals for up to 72 h of cultivation in complex medium (NB) using PCR. **G)** Expression of the terpene cyclase in *S. p.* 4Rx13 *cya*::Km and *crp*::Km in comparison to the wild type. Northern blot of *S. p.* 4Rx13 wild type, *cya*::Km and *crp*::Km was performed after 24 h cultivation in complex medium (NB). Detection of the terpene cyclase mRNA (upper panel) was conducted using hybridization with a DIG-labelled probe followed by fluorescence measurement for 1 min. 16 S rRNA detection (lower panel) was performed on the same blot as a positive control.

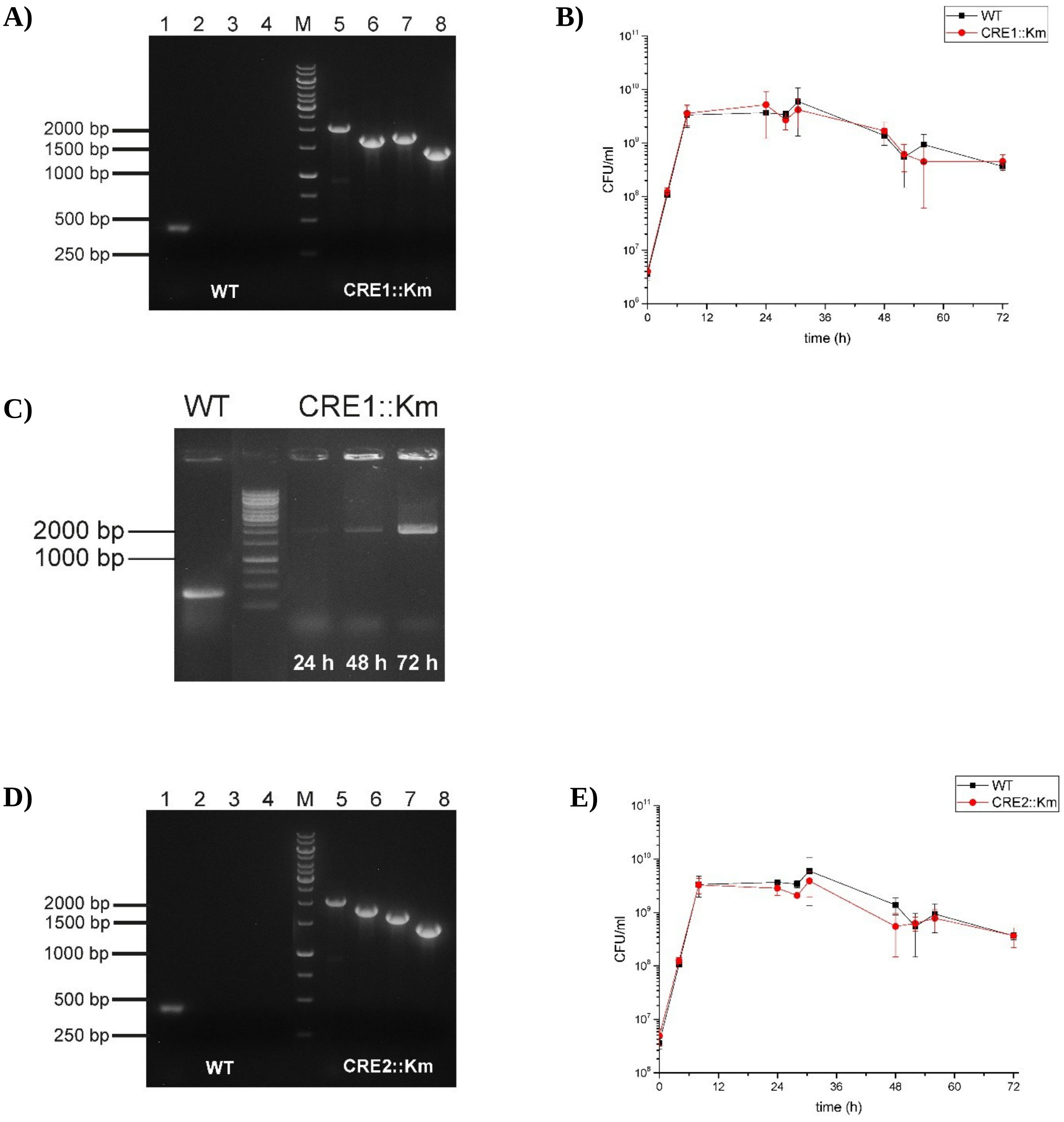

Figure S5: Characterization of the CRE1::Km and CRE2::Km deletion mutants of *Serratia plymuthica* 4Rx13. A)-C) Characterization of CRE1::Km. **A)** Verification of correct insertion of the functional cassette by PCR. In lanes 1-4 results for the *S. p.* 4Rx13 wildtype are shown and in lanes 5-8 for the CRE1 mutant. Lanes 1 & 5 represent amplification products with specific primers for the 5'-UTR of the sodorifen cluster (434 bp for the WT; 2.1 kb for CRE1::Km). Lanes 4 & 8 depict amplification products of primers specific for the functional cassette (1.4 kb). Lanes 2 & 6 show PCR products after amplification with 5'-UTR- and cassette-specific primers (1.68 kb; left border), as well as lanes 3 & 7 (right border; 1.8 kb). M = marker (GeneRuler® 1 kb). **B)** Growth comparison of *S. p.* 4Rx13 WT and CRE1::Km in complex medium (NB). The living cell number (CFU/ml) was determined periodically for up to 72 h. Error bars represent standard deviation (n=3). **C)** Stability of the deletion of CRE1 in *S. p.* 4Rx13 was determined in 24 h intervals for up to 72 h of cultivation in complex medium (NB) using PCR. **D)-E)** Characterization of CRE2::Km. **D)** Verification of correct insertion of the functional cassette by PCR. In lanes 1-4 results for the *S. p.* 4Rx13 wildtype are shown and in lanes 5- 8 for the CRE2 mutant. Lanes 1 & 5 represent amplification products with specific primers for the 5'- UTR of the sodorifen cluster (434 bp for the WT; 2.1 kb for CRE2::Km). Lanes 4 & 8 depict amplification products of primers specific for the functional cassette (1.4 kb). Lanes 2 & 6 show PCR products after amplification with 5'-UTR- and cassette-specific primers (1.8 kb; left border), as well as lanes 3 & 7 (right border; 1.66 kb). M = marker (GeneRuler® 1 kb). **E)** Growth comparison of *S. p.* 4Rx13 WT and CRE2::Km in complex medium (NB). The living cell number (CFU/ml) was determined periodically for up to 72 h. Error bars depict standard deviation (n=3).

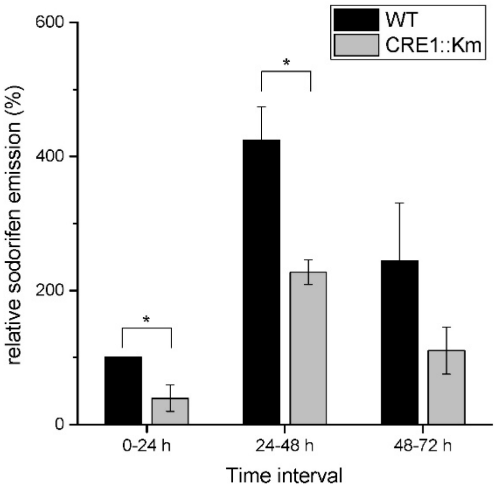

Figure S6: Relative sodorifen emission of *S. ply.* CRE1::Km in complex medium. Sodorifen emission of *S.ply.* 4Rx13 wild type and CRE1::Km during cultivation in complex medium (NBII) was determined using the closed VOC collection system (modified after Kai et. al. 2010) with subsequent GC/MS analysis. Relative sodorifen emission was calculated by correlating the living cell number (CFU/ml) to the sodorifen amount measured. As a reference, wild type emission until 24 h cultivation was used (100 %). Error bars indicate standard deviation (n=3). $*p < 0.05$

Table S1: Primer sequences and annealing temperatures. Underlined parts of primers represent the part binding to the functional cassette.

Gene	Direction	Tm (°C)	Sequence (5' ⟶ 3')	function
**SOD_c01380 (*cya*)**	sense	65	GAGACTGGATGCGATCAACC	gene specific primers
	antisense	67	CCCGTTCGATCTTCCAGTTG	
	sense	62	CCCGCGTTTCAACGGGTATACAGTCTGCTGCCTACCTTACTGCACTACCAAATTAACCCTCACTAAAGGGCGG	primers for mutagenesis
	antisense	62	CAGGCGTGTAAAGGCAAACGCCGTGGGAACGTTACCGTTCAGATAGCCCTAATACGACTCACTATAGGGCTC	
**SOD_c43810 (*crp*)**	sense	71	TCTCGGCAAACCGCAAACAG	gene specific primers
	antisense	67	CGTGAACAACCGACGATCTG	
	sense	62	GCTCCGTCGCGGTGCTGATTAAAGATGAAGAGGGTAAAGAGATGATCCTGAATTAACCCTCACTAAAGGGCGG	primers for mutagenesis
	antisense	62	CTGCGCTCCTGGCCTTCTTCAAACAATCCAAGCTCGCCGATGAAATCCCCTAATACGACTCACTATAGGGCTC	
**5'UTR**	sense	64.5	GATCCATCCGATTTGACACC	5'-UTR specific primers
	antisense	65	ATTCGATTGAGCATTGGGTG	
**CRE1::Km**	sense	62	GGGTATCGGTGAGCACGTAATATCATTGGGCTAGCCGACCCCTTTGTTTCAATTAACCCTCACTAAAGGGCGG	primers for mutagenesis
	antisense	62	TGGGAGTTTTGCTAGGGTGCAACTATTTTTTGTTAAAGCCAGGCGATGGATAATACGACTCACTATAGGGCTC	
**CRE2::Km**	sense	62	ATTAAGTTAAACCATAAGTTAAAAATATATCACTCGCTGATACTTCATCAAATTAACCCTCACTAAAGGGCGG	primers for mutagenesis
	antisense	62	TGTCAAATTTTATTTAATGACGGTTTTTAAGAAAAATAAATTAATCATGATAATACGACTCACTATAGGGCTC	
**SOD_c20750 (terpene cyclase)**	sense	65.5	ATCCCTTCAACCGGCAGTAC	gene specific primers
	antisense	64	CTGAAGCGGTATAAGCCAGC	
**16 S rRNA**	sense	62	AGAGTTTGATCATGGCTCAGA	gene specific primers
	antisense	70	AGGTGATCCAACCGCAGGTTC	
**SOD_c38830 (GAPDH)**	sense	62	ACCTGTTGGACGCAGAGTAC	gene specific primers
	antisense	64	GAGCGATCAGATCCAGAACC	
**Primer name**				
**Q_O**	sense	61.5	CCAGTGAGCAGAGTGACG	primers for 5'-RACE-PCR
**Q_I**	sense	61	GAGGACTCGAGCTCAAGC	
**Q_T**	sense	48	CCAGTGAGCAGAGTGACGAGGACTCGAGCTCAAGCTTTTTTTTTTTTTTTTT	
**GSP1**	antisense	69	CTTCCTGTAAACGCCGCTGG	
**GSP2**	antisense	73	ATGGCCGCAGCAACTGTTGG	
**pJet1-FP**	sense	59	ACTACTCGATGAGTTTTCGG	Primers for sequencing (provided by GATC)
**pJet1-RP**	antisense	57.5	TGAGGTGGTTAGCATAGTTC	

Table S2: Protocol for Reverse Transcriptase PCR.

Component	Volume
RNA (DNaseI-digested)	10 µl
antisense Primer (10 pmol/µl)	2 µl
dNTPs (10 mM each)	1 µl
DEPC-H_2O	1.5 µl
5 min, 65 °C, 1 min on ice	
5x RT-buffer (Thermo Fisher Scientific, Waltham, USA)	4 µl
Ribolock (Thermo Fisher Scientific, Waltham, USA)	0.5 µl
Maxima Reverse Transcriptase (Thermo Fisher Scientific, Waltham, USA) or DEPC-H_2O (negative control)	0.4 µl
DEPC-H_2O	0.6 µl

Amplification conditions

30 min, 50 °C
5 min 85 °C
1 min on ice

Table S3: Protocol for DIG-labelling of Northern blot probes. X = annealing temperature specific for the primers used.

Component	Volume
10x Taq buffer	5 µl
sense Primer	2 µl
antisense Primer	2 µl
dATP, dCTP, dGTP (10 mM each)	each 1 µl
dTTP (1 mM)	6.5 µl
DIG-dUTP (Roche Diagnostics Deutschland GmbH, Mannheim, Germany)	1 µl
Taq polymerase	1 µl
DNA template	1 µl

Amplification conditions

2 min 95 °C	
30 s 95 °C	40 cycles
30 s x °C	40 cycles
1 min 72 °C	40 cycles
7 min 72 °C	
∞ 10 °C	
